# Ferroptosis vulnerability of enzalutamide resistant prostate cancer conferred by ACSL4 overexpression and GPX4 antagonism

**DOI:** 10.1038/s41419-026-08906-8

**Published:** 2026-05-29

**Authors:** Yi Zhou, Jiapeng He, Haozhe Zhang, Qiqi Wang, Zhaojun Yu, Yifan Zhang, Sangsang Li, Yitong Chen, Weiwei Zhou, Qianyu Xu, Yu Yin, Qiang Wei, Chris Soon Heng Tan, Ju Guo, Bin Fu, Hailiang Hu

**Affiliations:** 1 Department of Biochemistry, SUSTech Homeostatic Medicine Institute, School of Medicine, Southern University of Science and Technology, Shenzhen, China; 2 Department of Chemistry, College of Science, Southern University of Science and Technology, Shenzhen, China; 3 Department of Urology, The First Affiliated Hospital of Nanchang University, Nanchang, China; 4 Department of Urology, The People’s Hospital of Guangdong Province, Southern Medical University, Guangzhou, China; 5 Department of Pathology, The First Affiliated Hospital of Anhui Medical University, Hefei, China

**Keywords:** Cancer therapeutic resistance, Cancer metabolism, Cancer stem cells

## Abstract

Enzalutamide, as a second-generation anti-androgen agent, has been used to treat castration-resistant prostate cancer (CRPC) or metastatic castration-sensitive prostate cancer (mCSPC). However, enzalutamide resistance inevitably developed for most treated CRPC/mCSPC, and limited effective therapies are currently available for these enzalutamide-resistant prostate cancers. In this study, we utilize our established enzalutamide-resistant prostate cancer cell lines to reveal a vulnerability of these cancer cells to GPX4-targeted ferroptosis. Interestingly, the established enzalutamide-resistant prostate cancer cells are mixed populations that predominantly exhibit stem cell-like (SCL) and neuroendocrine-like (NEL) phenotypes and may reflect cellular heterogeneity during the development of enzalutamide resistance in prostate cancer. We further demonstrated that ACSL4, a long-chain fatty acid-CoA ligase, was upregulated by the JAK/STAT pathway in enzalutamide-resistant SCL/NEL cells, thereby facilitating tumor proliferation and metastasis while increasing sensitivity to ferroptosis. To antagonize the ACSL4-conferred ferroptosis risk, SCL/NEL cells upregulated GPX4 through AP-1 transcription complex to suppress ferroptosis and thus promoted the malignant progression of SCL/NEL cells. Notably, we characterized Auranofin, an anti-rheumatoid arthritis drug, as a ferroptosis inducer for these SCL/NEL cells in vitro and in vivo by targeting AP-1 and decreasing GPX4 expression, suggesting a new application for Auranofin in treating enzalutamide-resistant stem cell-like AP-1^High^ CRPC.

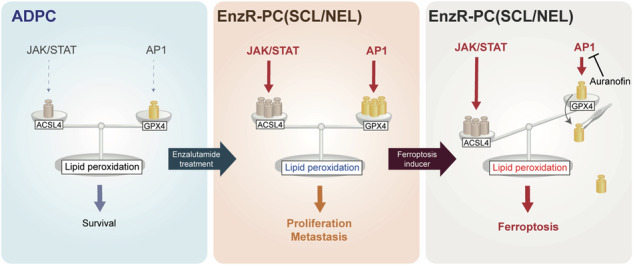

## Introduction

Anti-androgen therapy, including androgen-deprivation therapy (ADT) and the second-generation anti-androgen drugs such as enzalutamide, is the primary treatment choice for prostate cancer patients [1, 2]. However, castration resistance or enzalutamide resistance will inevitably develop, leading to the failure of anti-androgen therapy and the death of prostate cancer patients [3]. Cellular lineage plasticity has been reported to be a cellular mechanism mediating resistance to antiandrogen therapy [3, 4]. Androgen-sensitive prostate cancers are usually adenocarcinomas that express luminal lineage markers, while castration-resistant prostate cancer (CRPC) harbors mixed-lineage cells that emerge with antiandrogen therapy. Most recently, CRPC patients are classified into four types based on transcription factor expression: 1) CRPC-AR with AR and FOXA1; 2) CRPC-NE with NeuroD1 and ASCL1; 3) CRPC-SCL with AP-1/TEAD and 4) CRPC-WNT with TCF4 and LEF [5]. CRPC-NE, expressing neuroendocrine lineage markers such as *SYP*, *ENO2*, and *CHGA*, is the most studied type of lineage plasticity [6, 7]. ASCL1, N-Myc, BRN2, and ONECUT2 transcription factors, as well as RB1 and p53 deficiency, and epigenetic alterations such as EZH2 overexpression [4, 8], have been reported to drive the CRPC-NE transdifferentiation [9–12]. Chemotherapy is the first-line treatment for CRPC-NE patients, but with minimal benefits, and the survival rate is typically less than one year [13, 14]. CRPC-SCL, another large proportion of AR^-^/low CRPC patients, showed lower responsiveness to antiandrogen treatment [15]. In fact, the mechanistic study of lineage plasticity, as well as the treatment options for CRPC-SCL, are far less than those for CRPC-NE.

Ferroptosis is an iron-dependent form of cell death characterized by membrane lipid peroxidation and differs from apoptosis, necrosis, and pyroptosis [16, 17]. Ferroptosis is regulated by various proteins, iron ions, lipid ROS, and polyunsaturated fatty acids (PUFAs), among which ACSL4 is the first key rate-limiting enzyme for lipid peroxidation by activating the ferroptosis substrate PUFAs [18]. Cells develop a couple of mechanisms to suppress ferroptosis, including the GPX4-GSH pathway, the FSP1-CoQ pathway, the GCH1-BH4 pathway, and the DHODH-CoQH2 pathway [19]. These pathways suppress ferroptosis by scavenging membrane-bound free radicals and thereby maintaining membrane redox homeostasis [20–22]. The GPX4-GSH pathway is the primary mechanism for suppressing ferroptosis in most cells [19]. Several ferroptosis inducers have been developed to target the GPX4 pathway, including RSL3, erastin, fin56, and JKE-1674 [16, 23–25]. However, no ferroptosis inducer has been approved for clinical use currently. Interestingly, sorafenib, a RAF inhibitor, has been shown to induce ferroptosis by inhibiting the xCT system or other targets in hepatocellular carcinoma, renal carcinoma, and lung cancer cells [26–28], but not in different tumor cells [29]. Therefore, developing new clinically available ferroptosis inducers is also a medical urgency.

In this study, we established two enzalutamide-resistant prostate cancer cell lines in vitro. We found that these enzalutamide-resistant cells were a mixed population exhibiting stem cell-like (SCL) and neuroendocrine-like (NEL) phenotypes. We further demonstrated that these SCL/NEL cells were more sensitive to ferroptosis than their parental prostate cancer cells. Mechanistically, the activated JAK-STAT pathway in SCL/NEL cells induced the upregulation of ACSL4, which promotes cell proliferation and metastasis but also leads to ferroptosis potential. To evade ferroptosis, these SCL/NEL cells upregulated GPX4 by increasing AP-1 complex activity, thereby suppressing lipid peroxidation and maintaining a balance between ferroptosis and cell survival. Therefore, GPX4 inhibitors would disrupt the balance that leads to increased PUFA peroxidation in SCL/NEL cells. Interestingly, we found that the anti-rheumatoid drug, Auranofin, suppressed GPX4 expression to induce ferroptosis by inhibiting AP-1 transcriptional activity, providing a novel application of a clinically used drug to induce ferroptosis.

## Results

### Establishing an enzalutamide-induced SCL/NEL prostate cancer cell model

**Fig. 1:**
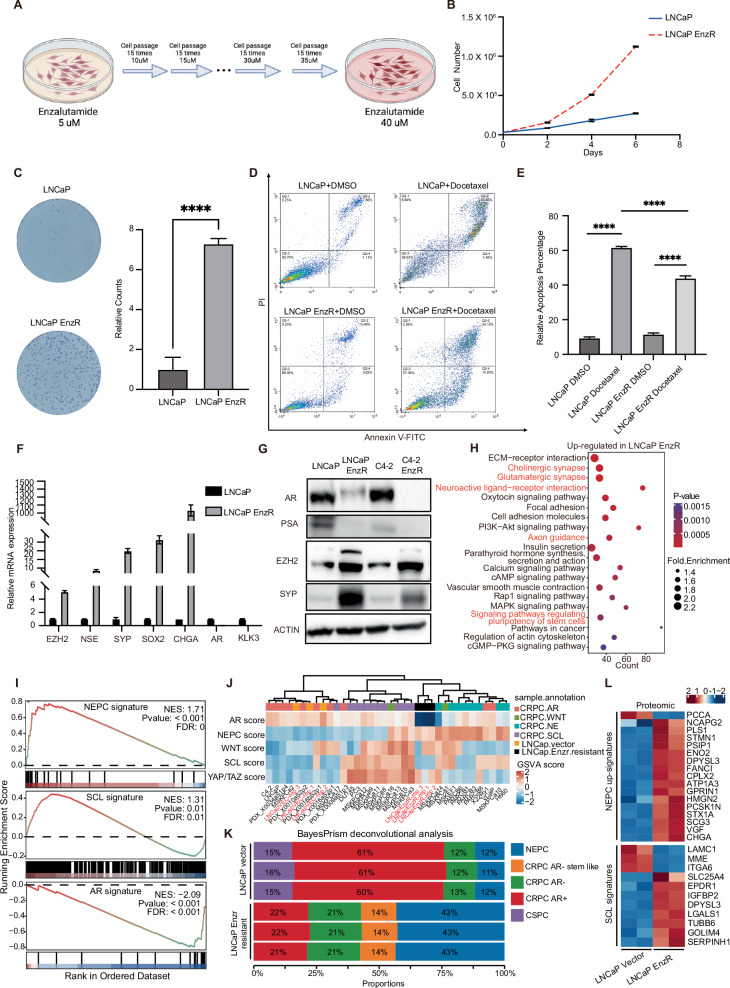
Establishing a treatment-induced stem cell-like (SCL)/neuroendocrine-like (NEL) prostate cancer cell model. **A** Establishment of enzalutamide-resistant LNCaP^EnzR^ cells and C4-2^EnzR^ cell lines via gradual dose escalation. The figure was created with BioRender.com (**B**). Cell number of LNCaP and LNCaP^EnzR^ (*n* = 3 parallel repeats). **C** Colony formation assay and relative quantification of LNCaP and LNCaP^EnzR^ Cells (*n* = 3 parallel repeats). **D**, **E** Apoptosis detection and quantitative analysis in LNCaP and LNCaP^EnzR^ cells treated with DMSO or Docetaxel (*n* = 3 parallel repeats). **F** RT-qPCR analysis of *EZH2, SOX2, NSE, SYP, CHGA, AR*, and *KLK3* mRNA expression in LNCaP and LNCaP^EnzR^ cells (*n* = 4 parallel repeats). **G** Western Blot Analysis of AR, PSA, EZH2, and SYP protein expression in LNCaP and LNCaP^EnzR^ cells. **H** KEGG Pathway Enrichment Analysis of differentially upregulated genes in LNCaP^EnzR^ cells. **I** Gene Set Enrichment Analysis reveals upregulation of NEPC and SCL signatures, accompanied by downregulation of AR signaling, in LNCaP^EnzR^ cells. **J** GSVA and Hierarchical Clustering of transcriptomic profiles in LNCaP and LNCaP^EnzR^ cells across published prostate cancer cohorts. **K** BayesPrism-based Deconvolution and Cellular Proportion quantification in LNCaP and LNCaP^EnzR^ transcriptomic data using scRNA-seq reference (GSE264573). **L** Proteomic Profiling reveals significant upregulation of NEPC and SCL protein signatures in LNCaP^EnzR^ cells. The experiments were repeated in three independent biological replicates. Data were shown as means ± s.d. and subjected to an Unpaired t-test, *p < 0.05, **p < 0.01, ***p < 0.001, ns means *p* > 0.05.

To study the molecular and metabolic mechanisms for enzalutamide resistance development of prostate cancer, we established two enzalutamide-resistant cell lines, LNCaP^EnzR^ and C4-2^EnzR^, by treating androgen-sensitive LNCaP cells and androgen-independent but enzalutamide-sensitive C4-2 cells with a series of concentrations of enzalutamide from 5 µM to 40 µM for 6-8 months (Fig. 1A). As expected, both LNCaP^EnzR^ cells and C4-2^EnzR^ cells were growing faster than control LNCaP and C4-2 cells (Fig. 1B and Fig. S1A). They had enhanced colony-forming capacity compared to parental control cells (Fig. 1C and Fig. S1B). Furthermore, we treated these cells with docetaxel and found that the enzalutamide-resistant cells exhibited greater resistance to docetaxel than their parental control cells (Figs. 1D, E, and Fig. S1C, D). We then examined adenocarcinoma, neuroendocrine, and stem cell markers in both parental control and enzalutamide-resistant cells. We found that the mRNA levels of neuroendocrine markers (*NSE*, *ENO2*, and *CHGA*) and stem cell markers (*EZH2* and *SOX2*) were up-regulated. In contrast, adenocarcinoma markers (*AR* and *KLK3*) were downregulated in both LNCaP^EnzR^ and C4-2^EnzR^ cells compared to their parental control cells (Fig. 1F and Fig. S1E). Western blotting further confirmed the high protein expression of the neuroendocrine marker SYP and the stem cell marker EZH2, as well as the low expression of the adenocarcinoma markers AR and KLK3 in LNCaP^EnzR^ cells and C4-2^EnzR^ cells (Fig. 1G). These findings suggest that LNCaP^EnzR^ and C4-2^EnzR^ cells exhibit greater malignancy and are more similar to stem cells and NE cells than LNCaP and C4-2 cells.

To further characterize the enzalutamide-resistant cells, we performed RNA sequencing and proteomic analysis for LNCaP^EnzR^ and LNCaP control cells. The RNA-Seq data revealed that 4,394 genes were significantly altered after the development of enzalutamide resistance (Fig. S1F). Principal component analysis (PCA) of gene expression profiles indicated that LNCaP^EnzR^ cells were different from LNCaP cells (Fig. S1G), and KEGG pathway analysis revealed neuron-related and stem cell-related pathways were upregulated in LNCaP^EnzR^ cells (Fig.1H). The cellular component and biological process analysis also revealed the enrichment of neuron-related pathways in LNCaP^EnzR^ cells (Fig. S1H, I). The gene set enrichment analysis (GSEA) also illustrated the increase of NEPC and SCL signature in LNCaP^EnzR^ cells and the decrease of AR signature (Fig. 1I). The Gene Set Variation Analysis (GSVA) and hierarchical clustering analysis of prostate cancer sequencing data showed that LNCaP cells were located in the CRPC/AR cluster. In contrast, LNCaP^EnzR^ cells were located in the CRPC/SCL and CRPC/NE clusters (Fig. 1J). Additionally, BayesPrism deconvolutional analysis was performed based on GSE264573 (Fig. S1J). The results illustrated the subpopulation changes from AR-dependent cell types CSPC (15%-16% in LNCaP/Vec to 0% in LNCaP^EnzR^) and CRPC/AR^+^ (60%-61% in LNCaP/Vec to 21%-22% in LNCaP^EnzR^) to AR-independent NEPC (11%-12% in LNCaP/Vec to 43% in LNCaP^EnzR^), CRPC/AR^-^ (12%-13% in LNCaP/Vec to 21% in LNCaP^EnzR^) and CRPC/AR^-^/stem cell-like (0% in LNCaP/Vec to 14% in LNCaP^EnzR^) during enzalutamide treatment (Fig. 1K). The proteomic data showed that neuroendocrine- and stem cell-related proteins were highly expressed in LNCaP^EnzR^ cells (Fig. 1L). These findings collectively suggest that stem cell and neuroendocrine cell differentiation occur during the development of resistance in LNCaP and C4-2 cells, and that LNCaP^EnzR^ cells and C4-2^EnzR^ cells exhibit mixed phenotypes, including stem cell-like and neuroendocrine-like prostate cancer cell phenotypes.

### SCL/NEL enzalutamide-resistant cells have enhanced GSH metabolism and are more sensitive to ferroptosis

**Fig. 2:**
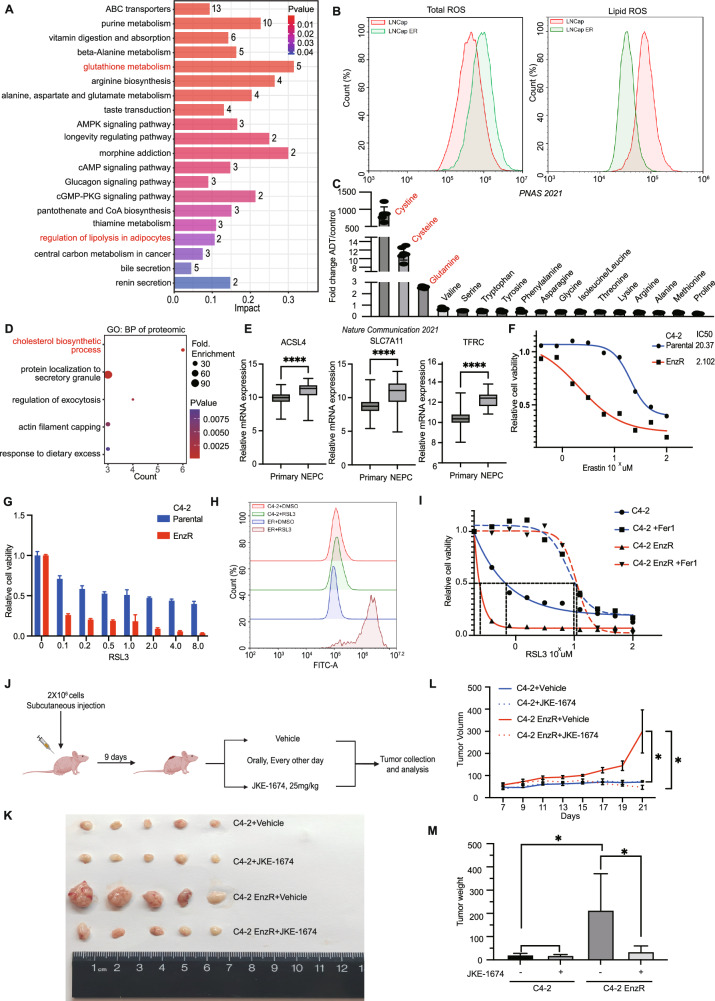
SCL/NEL prostate cancer cells have enhanced GSH metabolism and are more sensitive to ferroptosis. **A** Differential metabolite pathway enrichment analysis highlights key metabolic reprogramming in LNCaP^EnzR^ cells. **B** Detection of total Reactive Oxygen Species (ROS) and lipid peroxidation levels in LNCaP and LNCaP^EnzR^ cells (*n* = 3 parallel repeats). **C** Alterations in 17 amino acid levels in LNCaP cells with 14-day ADT treatment (*n* = 6 parallel repeats). **D** Biological Process Pathway Enrichment Analysis of differentially expressed proteins in LNCaP^EnzR^ cells. **E** Relative expression of *ACSL4, SLC7A11*, and *TFRC* in primary prostate cancer (*n* = 714) and NEPC (*n* = 19) samples. **F** Determination of IC₅₀ of the xCT inhibitor Erastin in C4-2 and C4-2^EnzR^ cells (*n* = 3 parallel repeats). **G** Dose-dependent effects of the GPX4 Inhibitor RSL3 on relative viability of C4-2 and C4-2^EnzR^ cells (*n* = 3 parallel repeats). **H** Detection of lipid peroxidation levels in C4-2 and C4-2^EnzR^ cells with the treatment of DMSO or RSL3 (*n* = 3 parallel repeats). **I** Measurement of relative cell viability treating C4-2 and C4-2^EnzR^ cells with RSL3 with or without ferrostatin-1 (2 μM) (*n* = 3 parallel repeats). **J** Schematic of subcutaneous tumor xenograft experiment using C4-2 and C4-2^EnzR^ cells in nude mice (*n* = 5 for each group). **K** Terminal tumor volumes in C4-2 and C4-2^EnzR^ subcutaneous xenografts. **L** Tumor volume over the course of treatment of C4-2 and C4-2^EnzR^ xenografts after three-week treatment with vehicle or JKE-1674. **M** Terminal tumor weights and statistical analysis of C4-2 and C4-2^EnzR^ xenografts. The experiments were repeated in three independent biological replicates. Data were shown as means ± s.d. and subjected to an Unpaired t-test, *p < 0.05, **p < 0.01, ***p < 0.001, ns means *p* > 0.05.

To explore the metabolic changes associated with the development of enzalutamide resistance, we conducted metabolite profiling of LNCaP and LNCaP^EnzR^ cells using mass spectrometry. We found that the heat map of metabolites illustrates a significant change in cellular metabolite profiles during the progression of enzalutamide resistance in LNCaP cells (Fig. S2D). Metabolic pathway analysis revealed a substantial enhancement in glutathione metabolism in LNCaP^EnzR^ cells (Fig. 2A), and a metabolomic volcano plot showed several metabolites related to glutathione metabolism were significantly elevated in LNCaP^EnzR^ cells (Fig. S2E). Given that glutathione metabolism is closely associated with reactive oxygen species (ROS), we first measured the intracellular ROS levels in these cells and found that the total ROS was higher in LNCaP^EnzR^ cells compared to LNCaP cells; however, the lipid ROS was lower in LNCaP^EnzR^ cells (Fig. 2B), suggestive of lower basic ferroptosis activity in LNCaP^EnzR^ cells. We have previously profiled metabolites in LNCaP cells treated with or without ADT for a short time (14 days) and found that polyunsaturated fatty acid (PUFA) levels were increased with ADT treatment [30, 31]. Actually, the levels of cystine, cysteine, and glutamine amino acid (all three contribute to the GSH biosynthesis) were significantly upregulated in ADT-treated LNCaP cells, which are more like NE-like cells (Fig. 2C). Importantly, pathway analysis of proteomic data showed that the cholesterol biosynthetic process was enhanced in LNCaP^EnzR^ cells (Fig. 2D and Fig. S2F), which is highly related to ferroptosis, suggesting that the enzalutamide resistance development may involve ferroptosis regulation. Additionally, bioinformatic analyses indicated that NEPC tumors significantly upregulated ferroptosis-related genes and those involved in ferroptosis suppression relative to primary prostate cancer and CRPC (Fig. 2E and Fig. S2G). These data suggest that SCL/NEL cells may successfully maintain lipid redox homeostasis, thereby avoiding ferroptosis and progressing to enzalutamide-resistant cells.

Alterations in GSH metabolism in LNCaP^EnzR^ cells may indicate greater susceptibility to ferroptosis. We treated these cells with erastin, an xCT inhibitor and a ferroptosis inducer. We found that the IC_50_ of C4-2^EnzR^ cells for erastin was significantly lower than that of C4-2 cells (Fig. 2F), and a similar trend was observed for LNCaP^EnzR^ cells, with the IC_50_ of LNCaP^EnzR^ cells being much lower than that of LNCaP (Fig. S2H). Furthermore, we treated the cells with another ferroptosis inducer, RSL3, and a GPX4 inhibitor, and found that both LNCaP^EnzR^ and C4-2^EnzR^ cells were more responsive to RSL3 than LNCaP and C4-2 cells (Fig. 2G and Fig. S2I). As ferroptosis is triggered by lipid peroxidation, we assessed lipid ROS levels after treating cells with RSL3. We found a significant increase in the lipid ROS level in LNCaP^EnzR^ and C4-2^EnzR^ cells, whereas no significant change was observed in the parental LNCaP and C4-2 cells (Fig. 2H and Fig. S2J). Additionally, we found that RSL3-induced cell death in all these cells could be rescued by Ferrostatin-1, a ferroptosis inhibitor (Fig. 2I and Fig. S2K). JKE-1674, another GPX4 inhibitor, demonstrates oral activity that induces cellular ferroptosis [32], and we observed a lower IC50 for JKE-1674 in C4-2^EnzR^ cells compared to C4-2 parental cells (Fig. S2L). We assessed the in vivo efficacy of JKE-1674 by treating C4-2 and C4-2^EnzR^ xenograft tumors via oral gavage (Fig. 2J). We found that C4-2^EnzR^ xenograft tumors grew more rapidly in vivo than C4-2 xenograft tumors, as expected. JKE-1674 significantly inhibited the growth of C4-2^EnzR^ tumors, whereas it had a minimal effect on the development of C4-2 tumors (Fig. 2K–M). Throughout the experiment, there were no substantial differences in body weight among the groups (Fig. S2M). Furthermore, since the SCL/NEL prostate cancer cell lines we established in vitro are based on AR signaling inhibition, we sought to determine whether the acquired ferroptosis sensitivity in SCL/NEL prostate cancer cells is caused by AR signaling suppression. We have established a series of LNCaP^EnzR^ cell lines that are resistant to varying concentrations of enzalutamide (5, 10, 30, 40 µM). We treated these LNCaP^EnzR^ cell lines and 40 µM enzalutamide-resistant C4-2^EnzR^ cells with RSL3 and measured malondialdehyde (MDA) levels, an intracellular lipid peroxidation marker. Compared with parental cells, basal ferroptosis was markedly elevated in 40 µM enzalutamide-resistant LNCaP^EnzR^ cells. Furthermore, RSL3-induced ferroptosis was significantly potentiated in 30 µM and 40 µM enzalutamide-resistant LNCaP^EnzR^ cells and in 40 µM enzalutamide-resistant C4-2 cells. Crucially, neither short-term androgen deprivation therapy (ADT) nor short-term enzalutamide treatment significantly increased RSL3-induced ferroptosis in LNCaP or C4-2 cells (Fig. S2N–S). These data indicate that prolonged adaptive resistance, rather than acute AR inhibition, confers heightened sensitivity to ferroptosis induction in these prostate cancer models. We also reanalyzed two independent clinical RNA-seq cohorts of prostate cancer, GSE126078 and the cohort reported by Westbrook et al. [33]. Across both datasets, *GPX4* expression showed no significant correlation with AR-activity scores/*AR* mRNA expression, indicating that the observed ferroptosis sensitivity phenotype is unlikely to be a direct consequence of AR inhibition (Fig. S2T–V). Taken together, these findings suggest that enzalutamide-resistant SCL/NEL prostate cancer cells are more susceptible to ferroptosis inducers than primary prostate cancer cells and CRPC cells, both in vitro and in vivo.

### ACSL4 upregulation leads to ferroptosis sensitivity as well as promotes the growth and migration of SCL/NEL prostate cancer cells

**Fig. 3:**
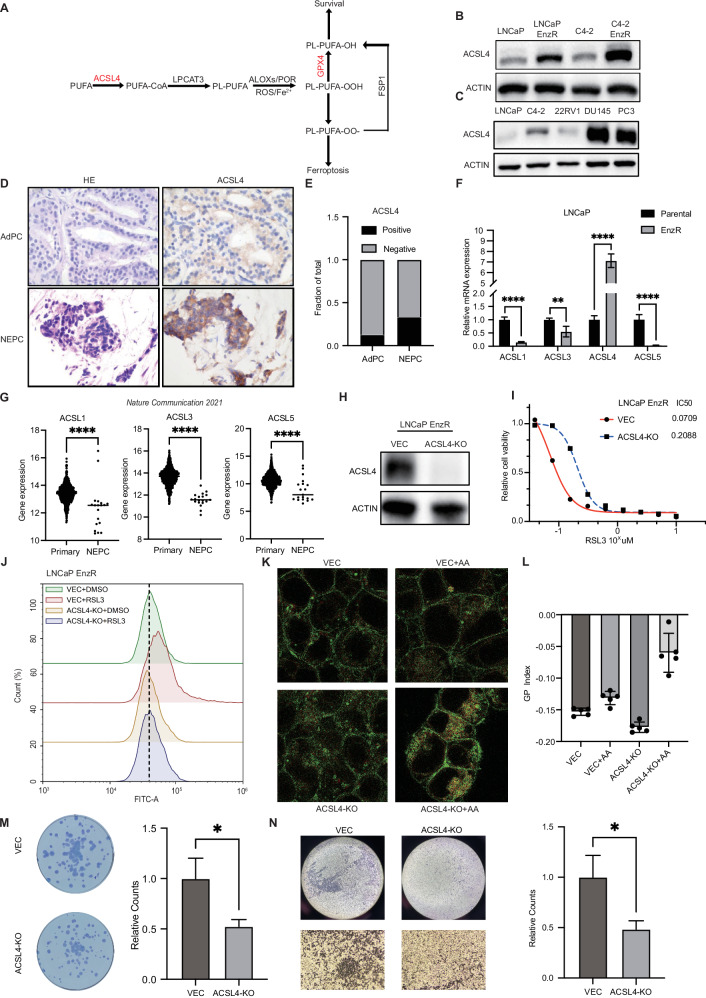
ACSL4 upregulation leads to ferroptosis sensitivity as well as promotes the growth and migration of SCL/NEL prostate cancer. **A** Mechanisms of Ferroptosis and Its Inhibition by GPX4 and FSP1. **B** Western Blot analysis of ACSL4 protein expression in LNCaP, LNCaP^EnzR^, C4-2, and C4-2^EnzR^ cells. **C** Western Blot Analysis of ACSL4 protein expression in LNCaP, C4-2, 22Rv1, DU145, and PC3 cells. **D** HE and IHC staining of ACSL4 protein expression in prostate adenocarcinoma and NEPC patient tissues. NEPC tissues are adenocarcinoma, and NEPC mixed tumor tissues with NE marker expression. Included 20 cases of adenocarcinoma (12 metastatic) and 13 cases of NEPC, among which 1 underwent orchiectomy, 1 received abiraterone combined with leuprolide and prednisone, 2 received bicalutamide combined with leuprolide, 2 were newly diagnosed, and 2 had no prior treatment history. The treatment history of the remaining 5 cases was not disclosed. **E** Quantitative analysis of IHC staining. **F** RT-qPCR analysis of *ACSL1, ACSL3, ACSL4*, and *ACSL5* mRNA expression in LNCaP and LNCaP^EnzR^ cells (*n* = 4 parallel repeats). **G** Relative expression of *ACSL1*, *ACSL3*, and *ACSL5* in primary prostate cancer (*n* = 714) and NEPC (*n* = 19) samples. **H** Western Blot analysis of ACSL4 protein expression in Vector and ACSL4 knockout LNCaP^EnzR^ cells **I** Determination of IC₅₀ of RSL3 in Vector and ACSL4 knockout LNCaP^EnzR^ cells (*n* = 3 parallel repeats). **J** Detection of lipid peroxidation levels in Vector and ACSL4 knockout LNCaP^EnzR^ cells with the treatment of DMSO or RSL3 (*n* = 3 parallel repeats). **K** Laurdan Staining Reveals Cell Membrane Polarity in C4-2^EnzR^/Vector and C4-2^EnzR^/sg-*ACSL4* Cells (*n* = 5 parallel repeats). **L** Quantitative analysis of Laurdan Staining. GP = (*I*440-*I*490)/(*I*440 + *I*490). *p* < *0.05*. **M** Colony formation assay and relative quantification of C4-2^EnzR^/Vector and C4-2^EnzR^/sg-*ACSL4* Cells (*n* = 3 parallel repeats). **N** Cell invasion assay and relative quantification of C4-2^EnzR^/Vector and C4-2^EnzR^/sg-*ACSL4* cells (*n* = 3 parallel repeats). The experiments were repeated in three independent biological replicates. Data were shown as means ± s.d. and subjected to an Unpaired t-test, *p < 0.05, **p < 0.01, ***p < 0.001, ns means *p* > 0.05.

The primary substrate of ferroptosis is PUFA, which is converted to PUFA-CoA and PL-PUFA by ACSL4 and LPCAT3 [34, 35]. PL-PUFA then undergoes peroxidation to produce the oxidized PL-PUFA-OOH that cause membrane structural instability and ultimately cell death (Fig. 3A). This process is primarily suppressed by the GPX4 pathway and FSP1 pathway [19], and ACSL4 is known as the initial rate-limiting enzyme at the onset of ferroptosis (Fig. 3A). The ferroptosis vulnerability of SCL/NEL cells prompts us to test whether high ACSL4 expression is in enzalutamide resistant prostate cancer cells, and found that the ACSL4 expression was significantly elevated in both LNCaP^EnzR^ and C4-2^EnzR^ cells compared to their parental cells (Fig. 3B). We further assessed the ACSL4 expression in a series of prostate cancer cells including LNCaP, C4-2, 22Rv1, DU145 and PC3, and found that LNCaP cells exhibited the lowest ACSL4 expression, while DU145 and PC3 cells displayed the highest ACSL4 expression (Fig. 3C), suggesting that more advanced prostate cancer cells express higher ACSL4 and may confer them more susceptible to ferroptosis. Furthermore, in vivo studies demonstrated that ACSL4 overexpression enhances tumor growth in xenografts (Fig. S2A–C). Actually, IHC staining showed that ACSL4 was highly expressed in NE-positive tumors compared to their adenocarcinoma counterparts (Fig. 3D, E).

ACSL4 is a member of the ACSL family responsible for PUFA esterification. At the same time, ACSL1, ACSL3, and ACSL5 are accountable for the esterification of saturated fatty acids (SFAs) and monounsaturated fatty acids (MUFAs) [36]. We evaluated the mRNA levels of the ACSL family members. We found that only *ACSL4* was significantly upregulated in LNCaP^EnzR^ tumors, whereas the other ACSL family genes, *ACSL1*, *ACSL3*, and *ACSL5*, were significantly downregulated in LNCaP^EnzR^ cells (Fig. 3F and Fig. S2D). Furthermore, bioinformatic analyses indicated that ACSL1, ACSL3, and ACSL5 were also downregulated. At the same time, *ACSL4* was upregulated in NEPC tumors compared to primary tumors (Figs. 3G, 2E and Figs. S3B, S2D), a finding similar to that observed in SCL/NEL cells.

To test whether ACSL4 overexpression contributes to the increased sensitivity of SCL/NEL cells to ferroptosis, we used the CRISPR/Cas9 system to knock out the ACSL4 gene in LNCaP^EnzR^ and C4-2^EnzR^ cells (Fig. 3H and Fig. S3C). We found that, compared to their parental control cells, both LNCaP^EnzR^-sg*ACSL4* and C4-2^EnzR^-sg*ACSL4* cells exhibited significantly increased resistance to the ferroptosis inducer RSL3 (Fig. 3I and Fig. S3D). Furthermore, we evaluated lipid peroxidation by BODIPY in control and *ASCL4* knockout cells with or without RSL3 treatment. We found that *ACSL4* knockout abrogated the RSL3-induced increase in lipid ROS in both LNCaP^EnzR^ and C4-2^EnzR^ cells (Fig. 3J and Fig. S3E), suggesting that ACSL4 overexpression mediates the increased ferroptosis sensitivity of enzalutamide-resistant prostate cancer cells.

The PUFA-to-MUFA ratio has been shown to affect the fluidity of the cell membrane [37], which may modulate the behavior of enzalutamide-resistant cells. To examine the effects of ACSL4 on cell membrane fluidity, we used Laurdan to stain cells with or without Arachidonic acid (AA) (a significant substrate for ACSL4), followed by calculation of the generalized polarization (GP) index. The GP index of C4-2^EnzR^ control cells did not change significantly with AA treatment. In contrast, the GP index of C4-2^EnzR^-sg*ACSL4* cells displayed a substantial increase with AA treatment, indicating that *ACSL4* knockout decreases the cell membrane’s fluidity (Fig. 3K, L). As a result, C4-2^EnzR^-sg*ACSL4* cells formed fewer colonies than control cells (Fig. 3M) and displayed a significantly reduced migration in comparison to the control cells (Fig. 3N). Taken together, ACSL4 overexpression in enzalutamide-resistant cells confers greater susceptibility to ferroptosis but simultaneously promotes aggressive cell growth and invasion.

### ACSL4 is upregulated by the JAK/STAT pathway in SCL/NEL prostate cancer cells

**Fig. 4:**
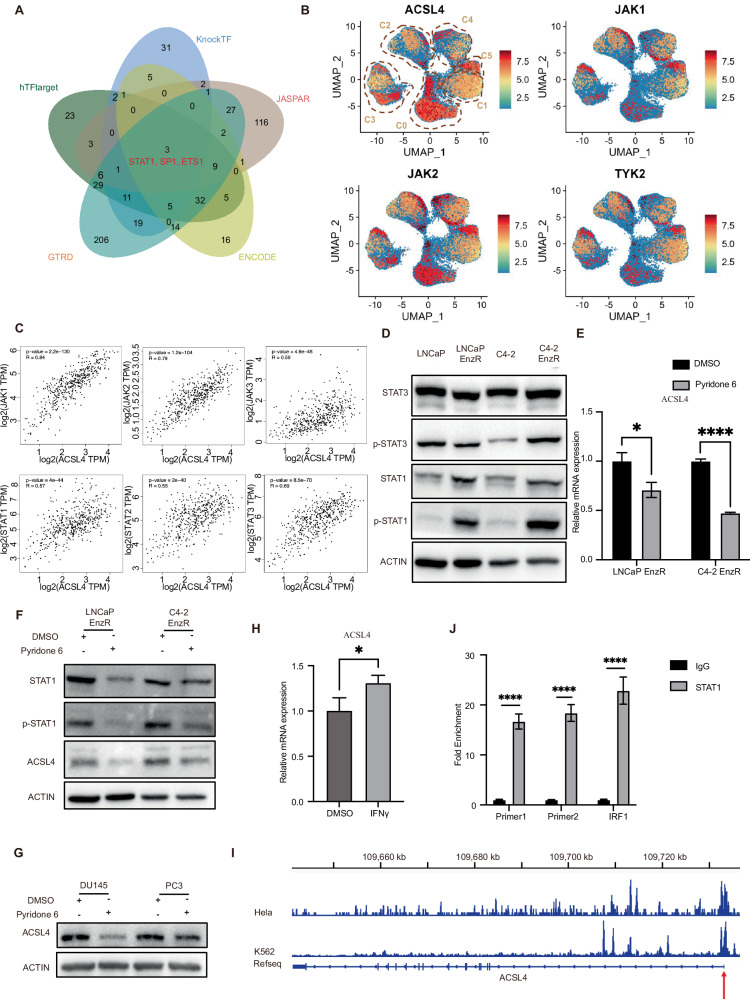
ACSL4 is upregulated by the JAK/STAT pathway in SCL/NEL prostate cancer cells. **A** Overlap of five transcription factor prediction tools. **B** Single-Cell RNA sequencing reveals stem-like and neuroendocrine-like prostate cancer subpopulations with *ACSL4, JAK1, JAK2, and TYK2* expression profiles. Data Source: Nature Cancer, Su Deng et al., 2022. **C** Correlation of *ACSL4* with *JAK1, JAK2*, *JAK3*, and *STAT1, STAT2*, *STAT3* expression in the TCGA PRAD Cohort (*n* = 497). **D** Western Blot analysis of STAT1, STAT3, p-STAT1, and p-STAT3 protein expression in LNCaP, LNCaP^EnzR^, C4-2, and C4-2^EnzR^ cells. **E** RT-qPCR analysis of *ACSL4* mRNA expression with DMSO or JAK inhibitor Pyridone6 treatment in LNCaP^EnzR^ and C4-2^EnzR^ cells (*n* = 4 parallel repeats). **F** Western Blot analysis of STAT1, p-STAT1, and ACSL4 protein expression with DMSO or Pyridone6 treatment in LNCaP^EnzR^ and C4-2^EnzR^ cells. **G** Western Blot analysis of ACSL4 protein expression with DMSO or Pyridone6 treatment in DU145 and PC3 cells. **H** RT-qPCR analysis of *ACSL4* mRNA expression with DMSO or IFNγ treatment in LNCaP^EnzR^ cells (*n* = 4 parallel repeats). **I** STAT1 binds the ACSL4 promoter, as revealed by chromatin immunoprecipitation sequencing. Data from Cistrome Data Browser. **J** ChIP-qPCR of STAT1 binding to the promoter of *ACSL4* and *CDK1* (positive control) in DU145 cells (*n* = 4 parallel repeats). The experiments were repeated in three independent biological replicates. Data were shown as means ± s.d. and subjected to an Unpaired t-test, *p < 0.05, **p < 0.01, ***p < 0.001, ns means *p* > 0.05.

The significant upregulation of *ACSL4* mRNA levels in enzalutamide-resistant prostate cancer cells may suggest that ACSL4 is regulated at the transcriptional level. To identify the transcription factor responsible for ACSL4, we first analyzed the ACSL4 promoter using five transcription factor prediction programs. We found three transcription factors (STAT1, SP1, and ETS1) with a high possibility to regulate *ACSL4* transcription (Fig. 4A). Given that the JAK/STAT pathway regulates the transformation of CRPC cells into stem-like cells and NEPC cells [38], we hypothesized that ACSL4 might be upregulated in SCL/NEL cells through the JAK/STAT pathway. Analysis of a single-cell RNA sequencing dataset revealed higher *ACSL4* expression in cells with high JAK family expression (Fig. 4B). Additionally, we conducted an analysis of the correlation between JAKs, STATs, and ACSL4 expression in the TCGA database. We found a positive correlation between the expression of *ACSL4* and *JAKs*, as well as a positive correlation between *ACSL4* and *STATs* expression (Fig. 4C). To further validate this correlation, we examined STAT1/3 and p-STAT1/3 in LNCaP, LNCaP^EnzR^, C4-2, and C4-2^EnzR^ cells. We found a significant elevation of STAT1 and p-STAT1 in LNCaP^EnzR^ and C4-2^EnzR^ cells compared to LNCaP and C4-2 cells (Fig. 4D). We then treated LNCaP^EnzR^ and C4-2^EnzR^ cells with JAK inhibitor Pyridone [6] and found a significant decrease in *ACSL4* mRNA and protein level, as well as in p-STAT1 level following JAKi treatment (Fig. 4E, F). A similar observation, that JAKi downregulated ACSL4, was also observed in prostate cancer cell lines DU145 and PC3 (Fig. 4G). On the other hand, the activation of JAK/STAT by IFNγ induced ACSL4 upregulation in LNCaP cells (Fig. 4H), suggesting that JAK/STAT activation does upregulate ACSL4. STAT1-ChIP sequencing has shown that STAT1 was able to bind to the promoter region of the *ACSL4* gene in K562 and HeLa cells (Fig. 4I). We employed ChIP-qPCR to confirm further that STAT1 acts as a transcription factor to bind to the promoter region of the *ACSL4* gene in C4-2^EnzR^ cells, with *IRF1* promoter serving as a positive control (Fig. 4J). Taken together, these data illustrated that a highly activated JAK/STAT pathway upregulated ACSL4 expression in SCL/NEL prostate cancer cells.

### GPX4 upregulation in SCL/NEL prostate cancer cells antagonizes ACSL4 overexpression-conferred ferroptosis vulnerability

**Fig. 5:**
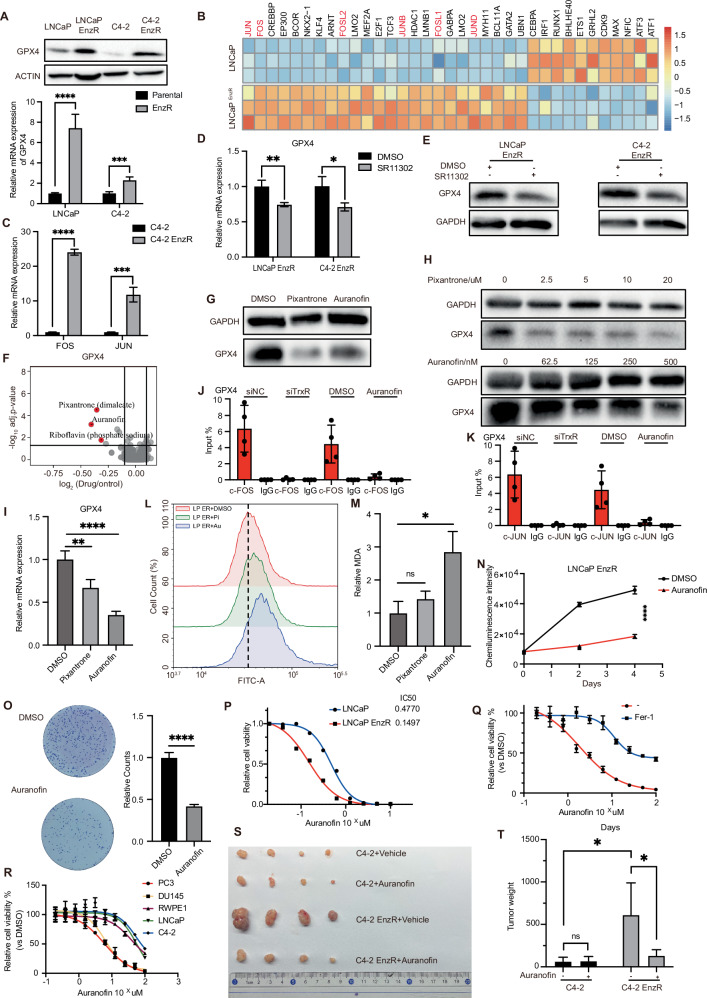
GPX4 upregulation in SCL/NEL prostate cancer cells antagonizes ACSL4 overexpression-conferred ferroptosis vulnerability, and Auranofin induces ferroptosis by targeting GPX4. **A** Western Blot and RT-qPCR analysis of GPX4 protein and mRNA expression in LNCaP, LNCaP^EnzR^, C4-2, and C4-2^EnzR^ cells (*n* = 4 parallel repeats). **B** Transcriptomic profiling identifies key transcription factors with altered expression. **C** RT-qPCR analysis of *FOS* and *JUN* mRNA expression in LNCaP, LNCaP^EnzR^, C4-2, and C4-2^EnzR^ cells (*n* = 4 parallel repeats). **D** RT-qPCR analysis of *GPX4* mRNA expression with DMSO or AP-1 inhibitor SR11302 treatment in LNCaP^EnzR^ and C4-2^EnzR^ cells (*n* = 4 parallel repeats). **E** Western Blot analysis of GPX4 protein expression with DMSO or SR11302 treatment in LNCaP, LNCaP^EnzR^, C4-2, and C4-2^EnzR^ cells. **F** TPP-MS Screening Identifies GPX4-Targeted Drugs. **G** Western Blot analysis of GPX4 protein expression with DMSO, Pixantrone, or Auranofin treatment in LNCaP^EnzR^ cells. **H** Auranofin and Pixantrone dose-dependently suppress GPX4 protein expression in LNCaP^EnzR^ cells. **I** RT-qPCR analysis of *GPX4* mRNA expression with DMSO, Pixantrone, or Auranofin treatment in LNCaP^EnzR^ cells (*n* = 4 parallel repeats). **J** ChIP-qPCR of FOS binding to the promoter of *GPX4* in DU145 cells with siNC, siTrxR, DMSO, and Auranofin (*n* = 4 parallel repeats). **K** ChIP-qPCR of JUN binding to the promoter of *GPX4* in DU145 cells with siNC, siTrxR, DMSO, and Auranofin (*n* = 4 parallel repeats). **L** Detection of lipid peroxidation levels with DMSO, Pixantrone, or Auranofin treatment in LNCaP^EnzR^ cells (*n* = 3 parallel repeats). **M** Detection of MDA levels with DMSO, Pixantrone, or Auranofin treatment in LNCaP^EnzR^ cells. **N** Relative cell viability of LNCaP^EnzR^ Cells with DMSO or Auranofin treatment (*n* = 3 parallel repeats). **O** Colony formation assay and relative quantification of LNCaP^EnzR^ Cells with DMSO or Auranofin treatment (*n* = 3 parallel repeats). **P** Determination of IC₅₀ of Auranofin in LNCaP and LNCaP^EnzR^ cells (*n* = 3 parallel repeats). **Q** The ferroptosis inhibitor Ferrostatin-1 (2 μM) partially rescued the cell death induced by Auranofin in LNCaP^EnzR^ cells. **R** Determination of the response rate of Auranofin in RWPE1, LNCaP, C4-2, PC3, and DU145 cells. **S** Terminal tumor volumes in C4-2 and C4-2^EnzR^ subcutaneous xenografts (*n* = 4 for each group). **T** Terminal tumor weights and statistical analysis of C4-2 and C4-2^EnzR^ xenografts. The experiments were repeated in three independent biological replicates. Data were shown as means ± s.d. and subjected to an Unpaired *t*-test, *p < 0.05, **p < 0.01, ***p < 0.001, ns means *p* > 0.05.

The upregulation of ACSL4 during enzalutamide treatment of prostate cancer cells renders them susceptible to ferroptosis; however, enzalutamide resistance still occurs in these cells, suggesting that enzalutamide-resistant cells develop a mechanism to evade ferroptosis. A recent study has shown that drug-resistant cancer cells were more dependent on GPX4, a major ferroptosis suppressor protein, to survive [39]. Therefore, we examined the GPX4 expression in these enzalutamide resistant cells and found that GPX4 expression was significantly upregulated in LNCaP^EnzR^ cells and C4-2^EnzR^ cells at both protein and mRNA levels (Fig. 5A), suggesting that GPX4 upregulation scavenges lipid free radicals caused by high ACSL4 expression and thus maintains the lipid redox balance of the membrane structure, preventing SCL/NEL prostate cancer cells from ferroptosis.

To investigate the mechanism of GPX4 upregulation, we analyzed transcription factor expression using RNA-seq data from LNCaP^EnzR^ versus LNCaP. We found that *FOS* and *JUN* (to form AP-1 transcription complex) were significantly upregulated in LNCaP^EnzR^ cells (Fig. 5B), which is further confirmed by RT-qPCR in C4-2^EnzR^ cells (Fig. 5C). This is consistent with the CRPC subtype classification that CRPC-SCL highly expressed AP-1 transcription factor [5]. To verify whether AP-1 regulates GPX4 expression, we first analyzed ChIP-seq data from the database and identified binding peaks for FOS and JUN in the GPX4 transcription start region (Fig. S4A). We then treated LNCaP^EnzR^ and C4-2^EnzR^ cells with the AP-1 inhibitor SR11302 and found that GPX4 expression was significantly downregulated in AP-1 inhibitor-treated cells compared to controls at both the RNA and protein levels (Fig. 5D, E). Simultaneously, we used siRNA to knock down *FOS* and *JUN* in PC3 cells, respectively. Under these conditions, we similarly observed a significant reduction in *GPX4* mRNA levels. It is worth noting that *ACSL4* mRNA levels remained unchanged, indicating that AP-1 knockdown does not affect ACSL4 expression (Fig. S4B). Taken together, these findings suggest that AP-1-mediated GPX4 upregulation suppresses ferroptosis conferred by ACSL4 overexpression, enabling SCL/NEL cells to escape ferroptosis.

### Auranofin induces ferroptosis by suppressing GPX4

Given that SCL/NEL cells are more dependent on GPX4 for maintaining membrane redox homeostasis, targeting GPX4 may offer a new therapeutic option for enzalutamide-resistant prostate cancers. Pixantrone and Auranofin were found to significantly affect the thermal stability of GPX4 by a TPP-MS (Thermal Proteomic Profiling Mass Spectrometry) [39, 40] (Fig. 5F). Furthermore, Pixantrone and Auranofin were confirmed to decrease the expression of GPX4 in LNCaP^EnzR^ cells (Fig. 5G). Interestingly, Auranofin, not Pixantrone, decreased the GPX4 expression in a dose-dependent manner (Fig. 5H). RT-qPCR showed that Pixantrone and Auranofin decreased *GPX4* mRNA level (Fig. 5I), but did not affect the half-life of GPX4 protein (Fig. S4C), suggesting that both compounds regulate GPX4 expression at the mRNA level.

The prostate cancer subtype CRPC-SCL has been shown to express the AP-1 transcription factor at a high level. We have now demonstrated that the AP-1 complex regulates the upregulation of GPX4 in enzalutamide-resistant SCL/NEL cells. Auranofin has been reported to inhibit TrxR (Thioredoxin Reductase), an enzyme that reduces thioredoxin, thereby affecting the DNA-binding affinity of transcription factors and regulating gene transcription [41–43]. Therefore, we wanted to test whether Auranofin inhibits GPX4 transcription by targeting AP-1. We constructed an AP1-luciferase reporter and found that Auranofin inhibited AP-1 transcriptional activity (Fig. S4D). To further clarify whether Auranofin affects AP-1’s DNA-binding capacity, we treated LNCaP^EnzR^, PC3, and DU145 cells with Auranofin; the latter two lines are considered typical SCL-CRPC cells. We then performed ChIP-qPCR to examine FOS and JUN binding at the *GPX4* promoter. As expected, Auranofin treatment significantly reduced AP-1 complex binding at the *GPX4* promoter, yielding a result similar to knocking down *TrxR* with siRNA (Fig. 5J, K and Fig. S4E–H). Taken together, our findings suggest that Auranofin induces ferroptosis by suppressing AP-1 transcriptional activity, which, in turn, decreases GPX4 transcription and consequently its protein expression.

We then tested whether Pixantrone or Auranofin induces ferroptosis in SCL/NEL cells. Although both Pixantrone and Auranofin induced an increase in lipid ROS (Fig. 5L and Fig. S4I) in LNCaP^EnzR^ and C4-2^EnzR^ cells, only Auranofin treatment significantly increased MDA (Fig. 5M and Fig. S4J). To test whether Pixantrone and Auranofin could reduce GPX4 expression in other tumor cells, we treated human chronic myelogenous leukemia cells (K562) and breast cancer cells (BT549) with Pixantrone and Auranofin. The results showed that Auranofin reduced GPX4 expression in both K562 and BT549 cells. At the same time, Pixantrone instead elevated GPX4 expression in K562 cells (Fig. S4K). Therefore, these findings suggest that Auranofin, but not Pixantrone, can induce ferroptosis in more tumors.

We then wanted to test whether Auranofin has anti-tumor effects. We found that Auranofin can inhibit the growth of both LNCaP^EnzR^ and C4-2^EnzR^ cells (Fig. 5N and Fig. S4L) and suppress their colony formation (Fig. 5O and Fig. S4M). Furthermore, knocking down *FOS* or *JUN* in PC3 cells produced cell growth inhibition effects similar to those of Auranofin treatment, further corroborating our previous findings (Fig. S4N). In addition, LNCaP^EnzR^ and C4-2^EnzR^ cells were more sensitive to Auranofin relative to LNCaP and C4-2 cells, in a similar way to RSL3 and JKE-1674 (Fig. 5P and Fig. S4O).

It is noted that treatment with ferroptosis inhibitors partially rescued the cell death and MDA level induced by Auranofin in vitro and in vivo (Fig. 5Q and Fig. S4P–W), further confirming the pharmacological role of Auranofin in inducing ferroptosis in prostate cancer cells. In fact, a previous study demonstrated, through in vivo experiments, that Auranofin induces ferroptosis without providing detailed mechanisms [44, 45]. Furthermore, compared with other prostate cancer cell lines, SLC-CRPC cells exhibit significantly greater sensitivity to Auranofin (Fig. 5R). Given the high AP-1 expression in SLC-CRPC, Auranofin may have potential as a specific therapeutic agent for SLC-CRPC.

We then performed xenograft experiments in Auranofin-treated animals. Given the sensitivity of enzalutamide-resistant prostate cancer cell lines to Auranofin in vitro, we administered Auranofin at a dose of 5 mg/mL to mice, as determined by other studies [46, 47]. We found that Auranofin significantly suppressed the growth of C4-2^EnzR^ xenograft tumors, with minimal effect on C4-2 xenograft tumor development (Fig. 5S, T).

## Discussion

**Fig. 6:**
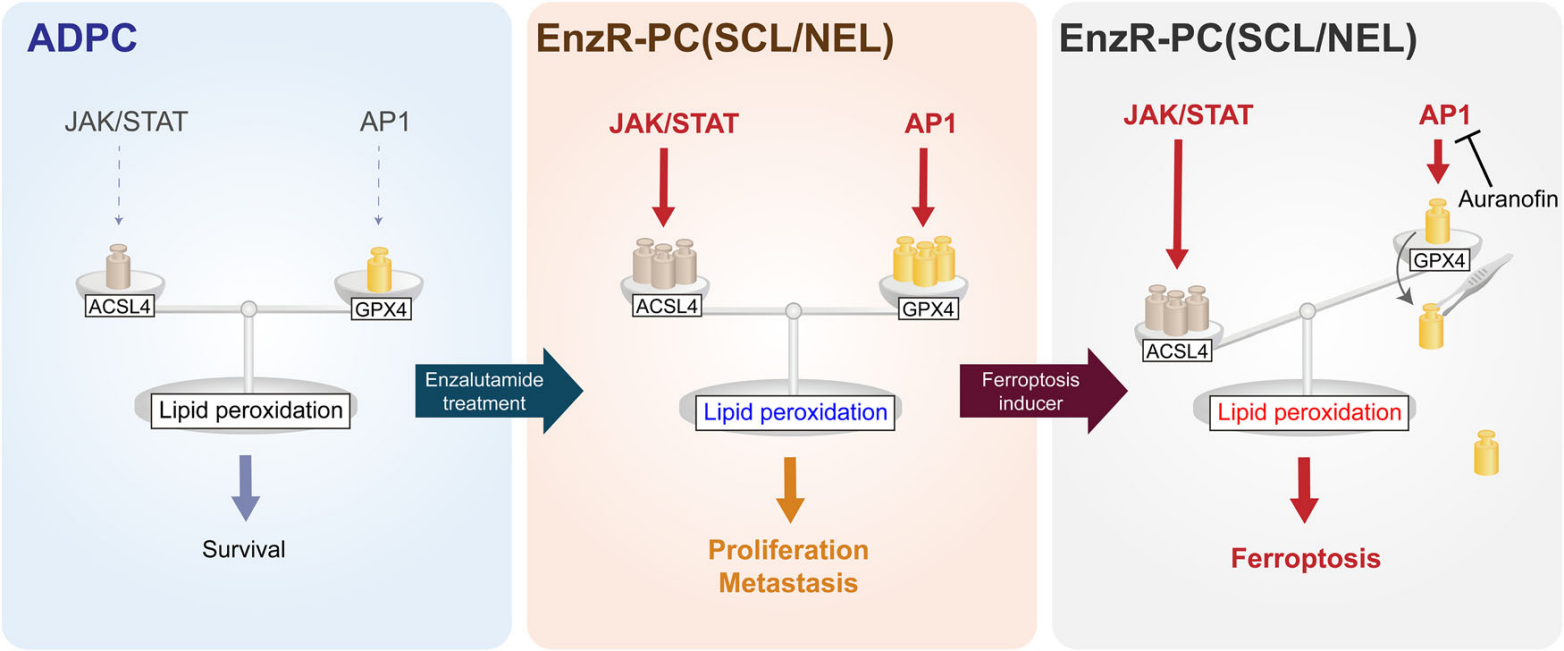
Working model. In prostate adenocarcinoma, ACSL4 and GPX4 are expressed at low levels, which helps maintain cell growth and proliferation. In enzalutamide-resistant SCL/NEL prostate cancer cells, the JAK/STAT pathway was highly activated, leading to increased ACSL4 expression and promoting tumor cell proliferation and metastasis. Concurrently, ACSL4 high expression induces ferroptosis vulnerability. To reduce the risk of ferroptosis, enzalutamide-resistant SCL/NEL cells upregulate GPX4 expression via the AP-1 transcription complex, thereby enhancing GSH metabolism to maintain redox homeostasis at the cell membrane. Auranofin disrupts the ACSL4/GPX4 balance and induces ferroptosis by reducing GPX4 expression through inhibition of AP-1 transcriptional activity to treat nzalutamide-resistant SCL/NEL prostate cancer.

In this study, we established in vitro cell models that mimic the enzalutamide-induced cellular heterogeneity. The resulting enzalutamide-resistant prostate cancer cells exhibited a mixed, multi-lineage phenotype, characterized by stem cell-like (SCL) and neuroendocrine-like (NEL) features. Using this model, we have demonstrated that the activated JAK/STAT pathway in SCL/NEL cells induces ACSL4 transcription, leading to its upregulation and promoting cell proliferation and tumor metastasis. Concurrently, ACSL4 overexpression renders cells vulnerable to ferroptosis. To counteract the risk of ferroptosis, enzalutamide-resistant SCL/NEL cells upregulate GPX4 expression through the AP-1 transcription complex, enhancing GSH metabolism and diminishing lipid peroxidation caused by ACSL4 overexpression. This allows enzalutamide-treated cells to escape ferroptosis and evolve into enzalutamide-resistant states. Importantly, we demonstrated that Auranofin, an FDA-approved drug for treating Rheumatoid Arthritis [48], can downregulate GPX4 expression by inhibiting AP-1 transcriptional activity and induce ferroptosis, thereby providing a new application for Auranofin to treat enzalutamide-resistant prostate cancer patients, especially those with CRPC-SCL (Fig. 6).

Prostate cancer is a disease with a relatively long course [49]. Anti-androgen therapy is the standard treatment for these patients [50]. Castration-resistant prostate cancer is highly aggressive, and the patient’s survival expectancy is extremely poor [51]. Currently, the treatment options for CRPC are minimal, and it is challenging to alleviate disease progression effectively [52]. There are many reasons for this phenomenon, and tumor heterogeneity cannot be ignored. In the evolution of prostate cancer, especially under treatment pressure, tumor cells often acquire lineage plasticity and differentiate into various cell subtypes [53]. Tumor heterogeneity among prostate cancer patients is a confirmed clinical fact, and the classification of CRPC has become more detailed [54]. On this basis, in recent years, intra-tumor heterogeneity (ITH) of prostate tumors has received increasing attention and is a possible cause of treatment resistance [55, 56]. Increasing evidence suggests that the lineage background of cancer cells within prostate tumors is essential. In treatment-induced tumor cell transdifferentiation, cells are at various stages of differentiation, and their biological characteristics, especially their response to different treatment regimens, may vary significantly [57]. The “mixed” characteristics of different subtypes of cancer cells in CRPC tumor masses have become a significant obstacle to treatment [58, 59]. Small cell carcinoma with AR signal loss and neuroendocrine characteristics (t-NEPC) is considered the most aggressive CRPC subtype [60]. It has become a hot topic in the field of basic research and treatment of prostate cancer in the past decade. Some t-NEPCs respond to chemotherapy drugs, but they will quickly develop drug resistance, which can eventually lead to the death of the patient [7].

Stemness is frequently used to characterize the poorly differentiated features of tumor cells, which exhibit transdifferentiation potential and strong therapeutic resistance [61]. Stem cell-like cellular subtypes have been identified in various tumor types [61]. Recent studies have indicated the presence of stem cell-like cellular subtypes in CRPC [5, 56]. It should be noted that tumor cells with stem cell-like characteristics are also intermixed in some patients clinically diagnosed with NEPC [57, 62]. SCL cells exhibit high differentiation potential at an early stage, but with stronger drug resistance [62]. Previously, CRPC-SCL was overlooked due to its relative scarcity. However, in our drug-resistant cell models derived from two different prostate cancer cell lines, neuroendocrine-like and stem cell-like differentiation co-occurred, suggesting that SCL differentiation is likely a common phenomenon in the progression and development of treatment resistance in prostate cancer. Alterations in key transcription factors and chromatin remodeling factors determine the direction of tumor cell transdifferentiation [5]. CRPC cell subtyping studies using clinical samples have shown that SCL-CRPC is regulated by AP-1 transcription complex [5], consistent with our experimental data. Previous studies have not provided direct therapeutic strategies targeting CRPC-SCL prostate cancer cells, and traditional anti-tumor drugs lack molecules that directly inhibit AP-1. Our research unexpectedly found that Auranofin inhibits AP-1 transcriptional activity in treating CRPC-SCL cells. Although initially approved only for treating rheumatoid arthritis, Auranofin has demonstrated significant potential for repurposing existing drugs in recent years [44, 45, 63, 64]. This stems from its pharmacologic action targeting TrxR, a key factor in intracellular redox regulation that is dysregulated in various cancers [65]. More importantly, TrxR activity is believed to influence the DNA-binding capacity of the AP-1 complex [66, 67], a pivotal transcription factor in SCL tumors [5]. Several studies have demonstrated Auranofin’s potential in cancer therapy [63, 64].

For instance, in tumors such as p53-mutated non-small cell lung cancer, Auranofin kills cancer cells by disrupting the cellular redox balance through targeting TrxR, and this disruption is closely linked to the GPX4-dependent system [63]. Related studies have identified Auranofin’s inhibitory effect on GPX4, though the specific mechanism remains unclear [64]. Additionally, studies have identified the regulatory role of the AP-1 transcription complex in GPX4 expression [68]. In this study, we demonstrated that AP-1 directly regulates GPX4 expression in enzalutamide-resistant prostate cancer cells. We also clarified that Auranofin targets TrxR to disrupt AP-1’s transcriptional function, ultimately impairing the GPX4-dependent ferroptosis suppression system and inducing ferroptosis. The sequential TrxR-AP-1-GPX4 signaling axis expands the clinical application potential of Auranofin, which targets TrxR. This implies that Auranofin not only affects TrxR-dependent cells but also disrupts the function of the key transcription factor AP-1, which provides a promising new clinical approach for the precision treatment of AP-1-high stem cell-like cancers.

Therapy resistance is a significant challenge in the treatment of cancers [69–71]. The high expression of GPX4 in enzalutamide-resistant SCL/NEL prostate cancer cells suggests that these cells may acquire a dependency on GPX4 and, therefore, may be more sensitive to GPX4 inhibitors. This dependence on GPX4 may arise from lineage conversion induced by prolonged drug stress, as the JAK/STAT signaling pathway is significantly activated during prostate cancer drug resistance and transdifferentiation [72, 73]. Increasing evidence indicates that JAK/STAT signaling not only influences cellular immune responses but also activates ACSL4 expression in immune cells and intestinal epithelial cells [74, 75]. This leads to altered lipid metabolism. It is important to note that high ACSL4 expression not only increases the risk of ferroptosis in cells but also promotes tumor cell metastasis both in vivo and in vitro [32, 76–78]. Our study extends this pathway to prostate cancer, suggesting that prostate cancer cells with highly activated JAK/STAT signaling may exhibit heightened sensitivity to ferroptosis induction. Although many GPX4 inhibitors have been reported to induce ferroptosis, none of them has been approved for clinical application [19]. Therefore, GPX4 is a promising target for treating tumor drug resistance [79]. However, the study of drugs targeting GPX4 remains unclear, although sorafenib can induce ferroptosis by targeting xCT in certain tumors [80].

Coordinating with our results, stem cell–like CRPC highly expressed AP-1 complex [5], and lineage plasticity is also regulated by AP-1 [81]. Therefore, AP-1 may be a favorable target of CRPC-SCL. In this study, we found that Auranofin can inhibit GPX4 expression and further demonstrated that it induces ferroptosis by suppressing AP-1 transcriptional activity, which is responsible for *GPX4* transcription. Therefore, our study suggests a new application of Auranofin to induce ferroptosis, which may be specific for treating AP-1-high CRPC-SCL tumors.

## Materials and methods

### Cell culture

All cell lines were obtained from the Cell Bank of the Chinese Academy of Sciences (Shanghai, China). LNCaP, LNCaP^EnzR^, C4-2, C4-2^EnzR^, 22Rv1, DU145, and PC3 cells were cultured in RPMI-1640 medium (Gibco, cat. #11875119), while HEK 293 T cells were cultured in DMEM medium (Gibco, cat. #10569044). Both media were supplemented with 10% foetal bovine serum (FBS, ExCell, cat. #FCS500) and 100 units/mL penicillin/streptomycin (Gibco, cat. #15140122). All the cells were maintained at 37°C with 5% CO_2_.

### Cell viability assay and colony formation assay

Cells were seeded in 96-well plates (2500 cells per well). After 24 hours, cells were treated with different drugs and DMSO. Cell viability was assessed using the CellTiter-LumiTM Luminescent Cell Viability Assay Kit (Beyotime, Shanghai, China). 500 cells were seeded in a 6-well plate and placed in a cell culture incubator for 10 days. The cells were then washed with PBS, fixed, and stained.

### RNA-seq and metabolite mass spectrometry

Cells were placed in 10 cm plates. When the cells reach 80% confluency, collect the cells for RNA-seq and metabolite mass spectrometry analysis. RNA sequencing was performed in collaboration with Haplox, and metabolite mass spectrometry was performed in collaboration with Panomix.

### Quantitative real-time PCR

RNA was extracted using FastPure Cell/Tissue Total RNA Isolation Kit (Vazyme, cat. # RC112). cDNA synthesis was performed by PrimeScript RT Master Mix HiScript IV All-in-One Ultra RT SuperMix for qPCR (Vazyme, cat. # R433). Quantitative real-time PCR (qRT-PCR) was applied on ABI 7500 (Applied Biosystems Co.) using AceQ qPCR SYBR Green Master Mix (Vazyme, cat. #Q111-02). GAPDH expression assay was used as an internal control. All qPCRs were determined in triplicate or more.

### Western blotting

Protein samples were extracted from cultured cells using RIPA lysis buffer (Beyotime, cat. #P0013C) supplemented with protease and phosphatase inhibitors (Thermo Scientific, cat. #78440). The protein concentration was determined using a BCA protein assay kit (Thermo Scientific, cat. #23236) according to the manufacturer’s instructions. Equal amounts of protein (20-30 µg) were separated by 10% SDS-polyacrylamide gel electrophoresis (SDS-PAGE) and subsequently transferred onto polyvinylidene difluoride (PVDF) membranes. The membranes were blocked with 5% non-fat milk in Tris-buffered saline containing 0.1% Tween-20 (TBST) for 1 hour at room temperature. After blocking, the membranes were incubated overnight at 4 °C with primary antibodies diluted in blocking buffer. Following three washes with TBST, the membranes were incubated with horseradish peroxidase (HRP)-conjugated secondary antibodies for 1 hour at room temperature. Protein bands were visualized using enhanced chemiluminescence (ECL) substrate and detected with a chemiluminescence imaging system.

### Tissue IHC staining

Human cancer tissue samples were obtained from The First Affiliated Hospital of Anhui Medical University. All tissue samples were collected in compliance with the informed consent policy. Fresh tumor tissue was reconstituted overnight in 10% neutral buffered formalin, washed once with PBS, and stored at 4 °C in 70% ethanol. Tissues were dehydrated and embedded in paraffin according to standard protocols. Embedded tissues were sectioned at a thickness of 5 μm for hematoxylin and eosin or IHC analysis. The slides were incubated overnight in a humidified chamber with the indicated primary antibody after boiling in 10 mM sodium citrate, pH 6.0, and incubating with 3% H_2_O_2_. Staining was performed using a DAB peroxidase substrate kit. Images were randomly captured at ×50 magnification using a microscope and scored for immunoreactivity.

### Total ROS and lipid peroxidation measurement

According to the manufacturer’s instructions, 1 × 10^5^ cells were seeded into each well of the 6-well plates. After 24 hours, the cells were treated with 2 μM RSL3 or DMSO for an additional 12 hours, then collected and washed with PBS. The cellular total ROS was then detected using the Reactive Oxygen Species Assay Kit with CM-H2DCFDA (Beyotime, cat. S0035S#). For lipid peroxidation measurement, incubated in 5 μM BODIPY 581/591 C11 dye reagent (Invitrogen, cat. #D3861) in PBS buffer containing 5% FBS at 37 °C, 5% CO_2_ for 30 min. Total ROS and Lipid-ROS levels were analyzed by flow cytometry (Agilent Co.).

### Xenograft mouse model

Nude (nu/nu) mice were purchased from Gempharmatech Co. 2 × 10^6^ of C4-2 or C4-2^EnzR^ cells were inoculated subcutaneously into 6-week-old male nude mice. 25 mg/kg JKE-1674 (MCE, cat. # HY-138153), Auranofin (MCE, cat. # HY-B1123), 10 mg/kg Ferrostatin-1 (MCE, cat. # HY-100579), and the vehicle (20% SBE-β-CD in Saline and 10% DMSO) were administered orally once every other day. Five or four mice were used in each group. In the experiment, each group of mice was randomly assigned to different treatment groups. For the experimenters, the grouping of mice was blinded during administration. Body weight and tumor volume are monitored every other day. Tumour volume (mm^3^) = (length × width^2^) / 2.

### Cell invasion assay

Cell invasion assays were conducted using Transwell chambers (LABSELECT, cat. # 14341) with 8.0 µm pore polycarbonate membranes pre-coated with Matrigel (diluted 1:8 in serum-free medium) to simulate the extracellular matrix (Corning, cat. # 356230). The Matrigel was allowed to solidify at 37°C for 4 hours. Cells were harvested, resuspended in serum-free medium, and seeded into the upper chamber at a density of 1 × 10^5^ cells/well. The lower chamber was filled with complete medium containing 10% FBS as a chemoattractant. The plates were incubated at 37°C in a 5% CO_2_ atmosphere for 24-48 hours to allow cell invasion. After incubation, non-invaded cells on the upper surface of the membrane were carefully removed using a cotton swab. Invaded cells on the lower surface of the membrane were fixed with 4% paraformaldehyde for 15 minutes and stained with 0.1% crystal violet for 20 minutes at room temperature. The stained cells were imaged under an inverted microscope, and five random fields per well were counted for quantification. Data were expressed as the mean number of invaded cells per field. Each experiment was performed in triplicate and repeated at least three times independently.

### Generalized polarization measurement

Cells were seeded in 6-well plates and cultured until reaching 70-80% confluency. Before staining, cells were washed twice with phosphate-buffered saline (PBS) and incubated with 5 µM Laurdan (dissolved in DMSO and diluted in PBS) at 37°C for 30 minutes in the dark. After incubation, cells were washed twice with PBS to remove excess dye. Fluorescence intensity was measured using a fluorescence spectrophotometer or a confocal microscope, with excitation at 350 nm and emission spectra collected at 440 nm (ordered phase) and 490 nm (disordered phase). The GP value was calculated using the formula: GP = (I_440_-I_490_)/(I_440_ + I_490_).

### Chromatin immunoprecipitation-qPCR assay

Chromatin immunoprecipitation (ChIP) was performed by following the protocol of the Sonication Chip Kit (ABclonal, cat. #RK20258), according to the manufacturer’s instructions. Cells were fixed with 1% paraformaldehyde (PFA, Biosharp, cat. #BL908A) and then washed with cold PBS. The cells were then harvested and subjected to cellular and nuclear lysis. The entire atomic lysate was sonicated under optimal conditions to yield DNA fragments of 200–700 bp. Twenty-five microlitres of sheared lysate was aliquoted as input. Four hundred and seventy-five microlitres of the sheared lysate was subjected to immunoprecipitation by 3 h incubation at 4 °C of STAT1 Rabbit mAb (Cell Signaling Technology, cat. #9172), c-FOS Rabbit mAb (Cell Signaling Technology, cat. #2250), c-JUN Rabbit mAb (Cell Signaling Technology, cat. #9165) or IgG Rabbit mAb (Cell Signaling Technology, cat. #3900) control. The immunoprecipitated DNA and input DNA were purified and amplified by qRT-PCR with primers as follows: ACSL4: forward primer 1, 5′-GATGCAGTGAAGAGTGCTA-3′; reverse primer 1, 5′- ACTTGCATCTGTGTGTCAGT-3′; forward primer 2, 5′-AACCGCGTGCCCGCTAGC-3′; reverse primer 2, 5′- TCCGGGCGCGTCTTTTCC-3′; GPX4: forward primer 1, 5′- GCTCGTGTAATCCCAGCTACTC -3′; reverse primer 1, 5′- CCCAAGCCCCTACGCAGAAAGA -3′; forward primer 2, 5′- CCAAACCATCCATGACGCCTCT -3′; reverse primer 2, 5′- CCGCCTAGGTGCTTGGGATTTGT -3′

### Malondialdehyde (MDA) measurement

Cells were lysed using RIPA lysis buffer (0.1 mL of lysis buffer per 10^6^ cells), and the supernatant was collected after centrifugation. The supernatant lysate was then analyzed using the Lipid Peroxidation MDA Assay Kit (Beyotime, cat. #S0131S). Subsequently, the protein concentration in the lysate was measured. Based on the detected MDA concentration and protein concentration, the MDA content per milligram of protein in the lysate was calculated.

### Thermal proteomic profiling mass spectrometry (TPP-MS) screening

Thermal proteome profiling (TPP) assessed compound-induced changes in protein thermal stability. Cells at 80–90% confluency were treated with compounds or DMSO for 6–72 hours, harvested, washed, and suspended. Aliquots were heated at 37°C or 52°C for 3 minutes. Lysates were prepared, clarified by ultracentrifugation, and protein concentration was measured *via* BCA assay. After reduction, alkylation, tryptic digestion, and TMT labeling, the pooled peptides were analyzed by LC-MS/MS.

MS data were processed (FDR < 1%) to generate thermal melt curves and calculate melting temperatures (Tm). ΔTm values (positive indicating stabilization, negative indicating destabilization) were computed, with significance assessed via t-tests and multiple comparisons correction (FDR < 0.05).

### siRNA transfection

To investigate gene function, siRNA-mediated knockdown was performed. Briefly, cells were seeded in complete growth medium without antibiotics in a culture plate to achieve 60-70% confluency at the time of transfection. For each transfection, a complex was prepared by diluting a specific siRNA targeting the gene of interest and the appropriate volume of Lipofectamine RNAiMAX (Invitrogen, #13778030) transfection reagent in separate tubes containing Opti-MEM Reduced Serum Medium. After a 5-minute incubation at room temperature, the diluted siRNA was combined with the diluted transfection reagent. The mixture was incubated for an additional 20 minutes to allow for the formation of siRNA-lipid complexes. This complex was then added dropwise onto the cells. The cells were incubated with the complexes at 37 °C, 5% CO_2_. Following an incubation period of 24 to 72 hours, the medium was replaced with fresh complete medium. The efficiency of gene knockdown was subsequently assessed by quantitative real-time PCR to analyze mRNA levels. A non-targeting scrambled siRNA was used in parallel as a negative control.

### Statistical analysis

All experiments were performed thrice or more, and data were presented as the mean ± standard deviation (s.d.). Statistically significant differences between the two groups were assessed using an unpaired t-test. *p* < 0.05 was considered significant. All statistical analyses were performed using GraphPad Prism software and Microsoft Excel.

## Supplementary information

Supplementary Figures

Supplementary table 1

Supplementary table 2

## Data Availability

All data generated or analyzed during this study are included in Supplementary Data. Table S1 shows differentially expressed genes in the transcriptome, and Table S2 shows differentially expressed proteins in the proteome. The RNA-seq datasets generated during this study are available in the Gene Expression Omnibus repository (PRJNA1266336). The raw TPP-MS screening data can be obtained by contacting the author (christan@sustech.edu.cn).
