# Supplementary material for: Ferroptosis vulnerability of enzalutamide resistant prostate cancer conferred by ACSL4 overexpression and GPX4 antagonism
Source: Cell Death Dis. 2026 May 29;17(1):665. doi: 10.1038/s41419-026-08906-8 (PMC13421459; doi:10.1038/s41419-026-08906-8)
Supplement: Supplementary file 1 — Supplementary Figures [file 41419_2026_8906_MOESM1_ESM.docx]

**SUPPLEMENTAL FIGURE LEGENDS**

**Supplemental Figure 1. Establishing SCL/NEL prostate cancer cell model** (A) Cell number of C4-2 and C4-2^EnzR^ (n = 3 parallel repeats). (B) Colony formation assay and relative quantification of C4-2 and C4-2^EnzR^ cells (n = 3 parallel repeats). (C and D) Apoptosis detection and quantitative analysis in C4-2 and C4-2^EnzR^ cells treated with DMSO or Docetaxel (n = 3 parallel repeats). (E) RT-qPCR analysis of *EZH2, SOX2, NSE, SYP, CHGA, AR*, and *KLK3* mRNA expression in C4-2 and C4-2^EnzR^ cells (n = 4 parallel repeats). (F) Volcano plot of differentially expressed genes (DEGs) between LNCaP and LNCaP^EnzR^ cells. (G) Principal Component Analysis (PCA) of LNCaP and LNCaP^EnzR^ cells. (H) Cellular component Pathway Enrichment Analysis of differentially upregulated genes in LNCaP^EnzR^ cells. (I) Biological process Pathway Enrichment Analysis of differentially upregulated genes in LNCaP^EnzR^ cells. The experiments were repeated in three independent biological replicates. Data were shown as means ± s.d. and subjected to an Unpaired t-test, *p < 0.05, **p < 0.01, ***p < 0.001, ns means p > 0.05.

**Supplemental Figure 2. SCL/NEL prostate cancer cells increased the sensitivity to ferroptosis inducers** (A) Terminal tumor volumes in C4-2 Plvx and C4-2 ACSL4^OE^ subcutaneous xenografts. (B) Tumor volume over day 11 to day 25 of C4-2 Plvx and C4-2 ACSL4^OE^ xenografts after injection. (C) Terminal tumor volumes and statistical analysis of C4-2 and C4-2^EnzR^ xenografts. (D) Heatmap of the Top 20 differential metabolites in LNCaP^EnzR^ cells. (E) Volcano plot of differential metabolites. Highlighting Glutathione Pathway Components. (F) Heatmap of differentially expressed proteins in the cholesterol biosynthetic process. (G) Relative expression of *ACSL4, SLC7A11,* and *TFRC* in CRPC (n = 316) and NEPC (n = 19) samples. (H) Determination of IC₅₀ of Erastin in LNCaP and LNCaP^EnzR^ cells (n = 3 parallel repeats). (I) Dose-dependent effects of the GPX4 Inhibitor RSL3 on relative viability of LNCaP and LNCaP^EnzR^ cells (n = 3 parallel repeats). (J) Detection of lipid peroxidation levels in LNCaP and LNCaP^EnzR^ cells with the treatment of DMSO or RSL3 (n = 3 parallel repeats). (K) Detection of lipid peroxidation levels in LNCaP and LNCaP^EnzR^ cells with the treatment of DMSO or RSL3 (n = 3 parallel repeats). (L) Determination of IC₅₀ of the JKE-1674 in C4-2 and C4-2^EnzR^ cells (n = 3 parallel repeats). (M) Mice weight over the course of treatment of C4-2 and C4-2^EnzR^ xenografts after three-week treatment with vehicle or JKE-1674. (N) Assessment of MDA levels in parental LNCaP cells and those adapted to various enzalutamide concentrations, with and without ferroptosis induction. (O) Measurement of MDA levels in LNCaP cells following short-term ADT or enzalutamide treatment, with and without ferroptosis induction. (P) Cell viability assay for LNCaP cells after short-term ADT or enzalutamide exposure. (Q) Assessment of MDA levels in parental C4-2 cells and an enzalutamide-resistant derivative, with and without ferroptosis induction. (R) Measurement of MDA levels in C4-2 cells following short-term enzalutamide treatment, with and without ferroptosis induction. (S) Cell viability assay for C4-2 cells after short-term enzalutamide exposure. (T) Comparison of GPX4 expression levels across five groups defined by AR status (AR-positive, AR-low, AR-negative) and neuroendocrine (NE) status (n = 97). (U) Scatter plot of GPX4 vs AR-signatures score in dataset GSE126078. (V) Scatter plot of GPX4 vs AR expression in the Westbrook *et al*. dataset (n = 42). The experiments were repeated in three independent biological replicates. Data were shown as means ± s.d. and subjected to an Unpaired t-test, *p < 0.05, **p < 0.01, ***p < 0.001, ns means p > 0.05.

**Supplemental Figure 3. High ACSL4 expression leads to ferroptosis sensitivity** (A) RT-qPCR analysis of *ACSL1*, *ACSL3*, *ACSL4*, and *ACSL5* mRNA expression in C4-2 and C4-2^EnzR^ cells (n = 4 parallel repeats). (B) Relative expression of *ACSL1*, *ACSL3,* and *ACSL5* in CRPC (n = 316) and NEPC (n = 19) samples. (C) Western Blot analysis of ACSL4 protein expression in Vector and ACSL4 knockout C4-2^EnzR^ cells. (D) Determination of IC₅₀ of RSL3 in Vector and ACSL4 knockout C4-2^EnzR^ cells (n = 3 parallel repeats). (E) Detection of lipid peroxidation levels in Vector and ACSL4 knockout C4-2^EnzR^ cells with the treatment of DMSO or RSL3 (n = 3 parallel repeats). The experiments were repeated in three independent biological replicates. Data were shown as means ± s.d. and subjected to an Unpaired t-test, *p < 0.05, **p < 0.01, ***p < 0.001, ns means p > 0.05.

**Supplemental Figure 4. High GPX4 expression in SCL/NEL prostate cancer cells decreased ACSL4 overexpression-induced lipid peroxidation, and Auranofin induces ferroptosis by targeting GPX4** (A). FOS and JUN bind to the GPX4 promoter, as revealed by ChIP-seq. Data from Cistrome Data Browser. (B) Following knockdown of FOS, JUN, and treatment with Auranofin in PC3 cells, mRNA levels of *FOS*, *JUN*, *GPX4*, and *ACSL4* were measured (n = 4 parallel repeats). (C) Detection of GPX4 protein half-life with the treatment of DMSO, Pixantrone, or Auranofin in LNCaP^EnzR^ cells. (D) Luciferase reporter assay of AP-1 transcription activity with DMSO or Auranofin treatment in LNCaP^EnzR^ and C4-2^EnzR^ cells (n = 3 parallel repeats). (E) ChIP-qPCR of FOS binding to the promoter of *GPX4* in LNCaP^EnzR^ cells with siNC, siTrxR, DMSO, and Auranofin (n = 4 parallel repeats). (F) ChIP-qPCR of JUN binding to the promoter of *GPX4* in LNCaP^EnzR^ cells with siNC, siTrxR, DMSO, and Auranofin (n = 4 parallel repeats). (G) ChIP-qPCR of FOS binding to the promoter of *GPX4* in PC3 cells with siNC, siTrxR, DMSO, and Auranofin (n = 4 parallel repeats). (H) ChIP-qPCR of JUN binding to the promoter of *GPX4* in PC3 cells with siNC, siTrxR, DMSO, and Auranofin (n = 4 parallel repeats). (I) Detection of lipid peroxidation levels with DMSO, Pixantrone, or Auranofin treatment in C4-2^EnzR^ cells (n = 3 parallel repeats). (J) Detection of MDA levels with DMSO, Pixantrone, or Auranofin treatment in C4-2^EnzR^ cells (n = 3 parallel repeats). (K) Western Blot analysis of GPX4 protein expression with DMSO, Pixantrone, or Auranofin treatment in K562 and BT549 cells. (L) Relative cell viability of C4-2^EnzR^ Cells with DMSO or Auranofin treatment (n = 3 parallel repeats). (M) Colony formation assay and relative quantification of C4-2^EnzR^ Cells with DMSO or Auranofin treatment (n = 3 parallel repeats). (N) Cell counts 48 hours after knocking down FOS and JUN or treating with Auranofin in PC3 cells (n = 3 parallel repeats). (O) Determination of IC₅₀ of Auranofin in C4-2 and C4-2^EnzR^ cells (n = 3 parallel repeats). (P) The ferroptosis inhibitor Ferrostatin-1 partially rescued the cell death induced by Auranofin in C4-2^EnzR^ cells (n = 3 parallel repeats). (Q) The ferroptosis inhibitor Ferrostatin-1 partially rescued the cell death induced by Auranofin in DU145 cells (n = 3 parallel repeats). (R) The ferroptosis inhibitor Ferrostatin-1 partially rescued the cell death induced by Auranofin in PC3 cells (n = 3 parallel repeats). (S) Terminal tumor volumes in C4-2^EnzR^ subcutaneous xenografts. (T) Mice’s weight over the course of treatment of C4-2^EnzR^ xenografts after two weeks of treatment with vehicle, Auranofin, or Auranofin with Fer-1. (U) Tumor volumes over the course of treatment of C4-2^EnzR^ xenografts after two weeks of treatment with vehicle, Auranofin, or Auranofin with Fer-1. (V) Terminal tumor volumes and statistical analysis of C4-2^EnzR^ xenografts. (W) Terminal tumor MDA level and statistical analysis of C4-2^EnzR^ xenografts. The experiments were repeated in three independent biological replicates. Data were shown as means ± s.d. and subjected to an Unpaired t-test, *p < 0.05, **p < 0.01, ***p < 0.001, ns means p > 0.05. The experiments were repeated in three independent biological replicates. Data were shown as means ± s.d. and subjected to an Unpaired t-test, *p < 0.05, **p < 0.01, ***p < 0.001, ns means p > 0.05.
